# Influencing Factors Regarding the Severity of Peri-Implantitis and Peri-Implant Mucositis

**DOI:** 10.3390/diagnostics14141573

**Published:** 2024-07-19

**Authors:** Csilla Benedek, Bernadette Kerekes-Máthé, Liana Bereșescu, Imola Zsuzsa Buka, Zsuzsanna Bardocz-Veres, Ildikó Geréb, Krisztina Ildikó Mártha, Kinga Mária Jánosi

**Affiliations:** 1 Department of Periodontology and Oral Diagnosis, “George Emil Palade” University of Medicine, Pharmacy, Science and Technology of Târgu Mureș, 540142 Târgu Mureș, Romaniaimola.buka@umfst.ro (I.Z.B.); 2 Department of Tooth and Dental Arch Morphology, “George Emil Palade” University of Medicine, Pharmacy, Science and Technology of Târgu Mureș, 540142 Târgu Mureș, Romania; 3 Department of Preventive and Community Dentistry, “George Emil Palade” University of Medicine, Pharmacy, Science and Technology of Târgu Mureș, 540142 Târgu Mureș, Romania; 4 Department of Prosthodontics and Oral Rehabilitation, “George Emil Palade” University of Medicine, Pharmacy, Science and Technology of Târgu Mureș, 540142 Târgu Mureș, Romania; zsuzsanna.bardocz-veres@umfst.ro; 5 Windisch Dental-Wident, 1053 Budapest, Hungary; gerebildiko23@gmail.com; 6 Department of Orthodontics, “George Emil Palade” University of Medicine, Pharmacy, Science and Technology of Târgu Mureș, 540142 Târgu Mureș, Romania; krisztina.martha@umfst.ro; 7 Department of Fixed Prosthodontics, “George Emil Palade” University of Medicine, Pharmacy, Science and Technology of Târgu Mureș, 540142 Târgu Mureș, Romania

**Keywords:** peri-implantitis, peri-implant mucositis, bone resorption

## Abstract

The scientific literature is increasingly focused on peri-implant mucositis and peri-implantitis, which are biological outcomes of dental implant treatment. Background/Objectives: The present study aimed to evaluate the two most critical complications of dental implantation, peri-implant mucositis and peri-implantitis, through the prism of different influencing factors. Methods: We followed 40 patients, with a total number of 92 dental implants, divided into three age groups: under 35 years, between 35 and 55 years, and older than 55 years. Patients were also divided into groups according to the time since implant placement: 1–3 years, 4–7 years, and more than 7 years. The patients were examined, and periodontal pocket depth, peri-implant pocket depth, Löe–Silness gingival index, mucosal thickness, and keratinized mucosal width were recorded; bone resorption was measured on radiographs using a 2D image analysis method; and a questionnaire was also conducted. Results: Bone resorption was highest in the 35–55 age group (3.09 ± 0.04 mm) and for implants placed 4–7 years ago (3.39 ± 0.12 mm). Females had a mean bone resorption of 3.4 ± 0.15 mm and males of 2.45 ± 0.07 mm. Statistically, there was a significant difference only in the Löe–Silness index: the 35–55 age group had the highest values (*p* = 0.04). Conclusions: There were no statistically significant differences between the time since implant placement and the degree of bone resorption, nor between sexes. Peri-implant inflammation may occur at any age, regardless of the lifetime of the implants.

## 1. Introduction

Dental implants have many advantages over the classic and traditional fixed partial dentures: a much higher success rate, as shown by the majority of the studies conducted in this field (over 97% success rate for ten years), better preservation of bone in the edentulous areas, a lower risk of caries formation, a reduction in adjacent natural teeth sensitivity, and less endodontic damage to neighboring teeth [1,2,3].

### 1.1. The Success Rate of the Implantation

The implant can be successful if it is stable during the examination and if the tissues surrounding it are inflammation-free [4].

The success rate depends, among other things, on primary stability, which means that the implant is firmly seated in the socket. Factors influencing primary stability include bone density, the size and character of the bone–implant interface, the implant’s shape, and the surgical technique [5,6,7].

The stability and overall health of the hard and soft tissues around implants, among other factors influencing the implant–prosthetic complex, are critical to the outcome of implant therapy [8,9,10]. The integrity of the hard and soft tissues surrounding a dental implant can be compromised by peri-implant diseases such as peri-implant mucositis and peri-implantitis, which are inflammatory lesions that develop due to plaque accumulation in the surrounding tissues [9]. 

### 1.2. Peri-Implant Mucositis

The 2017 World Workshop on the Classification of Periodontal and Peri-Implant Diseases and Conditions describes peri-implant mucositis as “a disease that involves inflammation of the soft tissues surrounding a dental implant without additional bone loss after the initial bone remodeling that may occur during healing after the surgical placement of the implant” [9,11,12,13]. 

Peri-implant mucosal inflammation and a lack of progressive marginal peri-implant bone loss are the primary diagnostic criteria for peri-implant mucositis [14,15]. Peri-implant mucositis is a reversible, treatable condition. It is thought to be a precursor to peri-implantitis. Once clinical signs of peri-implant mucositis are found during an exam, an X-ray that shows no bone loss confirms the diagnosis [14,16].

### 1.3. Peri-Implantitis

Peri-implantitis is a plaque-associated pathologic modification of the tissues surrounding the implant, characterized by inflammatory cells in the peri-implant mucosa and progressive bone loss around dental implants [9,12]. It is believed to be the primary factor in implant failure after osseointegration [9,17].

Sites of peri-implantitis show signs of mucosal inflammation surrounding the implant and/or gingival recession, accompanied by increased probing depths around implants due to radiologically detected bone loss. Suppuration of the peri-implant pockets indicates the active nature of the pocket [11]. In the absence of peri-implant probing depth values from a previous examination or in the absence of previous radiographs, which are the most important indicators, a positive diagnosis of peri-implantitis can be established based on the following: positive bleeding index on probing; 6 mm or more peri-implant probing depth values; and 3 mm or more marginal bone level apical to the most coronal area of the endosseous component of the implant [11,14].

The shape of bone resorption in peri-implantitis is circular around the implant. The development of bone loss is rapid, does not follow a linear pattern, and occurs during the first three years of use [14,18,19,20,21]. In this order, peri-implantitis causes accelerated and severe bone loss and subsequent implant failure and loss [22].

### 1.4. Risk Factors for Developing Peri-Implant Inflammations

Several risk factors, such as smoking, poor oral hygiene, prior periodontal disease, and lack of professional and personal maintenance, have been associated with an increased likelihood of developing peri-implantitis [9,23]. Notably, these elements are also recognized as a risk factor for developing periodontitis [9,24]. The relationship between peri-implantitis and periodontitis has been studied over time, and a strong link has been observed: both conditions are the result of bacterial inflammation [22,25].

Individuals who do not practice good dental hygiene have an odds ratio of 14.3, which increases their likelihood of developing peri-implantitis [9,24,26]. Inconsistent or nonexistent complementary supportive and maintenance therapy has also been shown to increase the risk of tooth loss and relapse of periodontal disease [27].

Smoking has direct and indirect effects on the periodontium by inhibiting cytokine production, altering humoral immune system function, impairing neutrophil activities, stimulating capillary vasoconstriction and fibrosis, and increasing the prevalence of periodontal pathogens in periodontal pockets [9,24]. These modifications may increase the subject’s risk of developing peri-implantitis in addition to making them more susceptible to periodontitis.

Local predisposing factors play a significant role in the prevalence of peri-implant conditions. Prosthetic parameters, including implant location, the type and pattern of prosthetic connection emergence, and residual cement in the tissues surrounding implants, have been shown to influence these peri-implant conditions [28].

The main objective of the present study was to evaluate the two most critical complications of dental implantation, peri-implant mucositis and peri-implantitis, through the prism of various influencing factors.

## 2. Materials and Methods

Forty patients were selected from cases treated at a dental clinic in Mures County between November 2022 and February 2024. Power calculations with G*Power version 3.1.9.6. software (Franz Faul, Universität Kiel, Kiel, Germany) determined the sample size calculation with 80% power, alpha = 0.05.

The following inclusion criteria were used:

- At least one dental implant placed.
- Absence of systemic diseases in the background, especially those that directly affect the success and survival rate of dental implants, such as osteoporosis, diabetes, neurological disorders, HIV, hypothyroidism, and cardiovascular diseases.
- Absence of oral cavity changes, such as autoimmune mucocutaneous diseases, necrotizing periodontal diseases, scurvy, and extensive necrosis in agranulocytosis.
- Absence of medication: Only patients who were not taking any medication were included in this study. The most important drugs that could affect the survival rate of dental implants are bisphosphonates, non-steroidal anti-inflammatory drugs (NSAIDs), glucocorticoids, proton pump inhibitors (PPIs), and serotonin reuptake inhibitors (SSRIs).
- Non-smoking patients.
- Good or acceptable patient oral hygiene protocol, performed at least twice daily. Oral hygiene habits were considered by designing a questionnaire.

An evaluative digital panoramic radiograph was taken during periodic checkups.

Exclusion criteria:

- No inserted dental implant;
- Presence of systemic diseases;
- Presence of oral cavity changes;
- Long-term daily medication of the patient;
- Smoking patients;
- Poor oral hygiene;
- Absence of evaluable digital panoramic X-rays.

Our study was approved by the Scientific Research Ethics Committee of the “George Emil Palade” University of Medicine, Pharmacy, Science and Technology of Targu Mures (1895/19 October 2022).

The gender distribution of the investigated cases: 21 women and 19 men. Cases were divided into three groups according to age: those under 35 years (group A), those between 35 and 55 years (group B), and those over 55 years (group C). The cases were also divided into three groups according to the time since implant placement: implants 1–3 years old (group 1), implants 4–7 years old (group 2), and implants older than 7 years (group 3).

**Figure 1:**
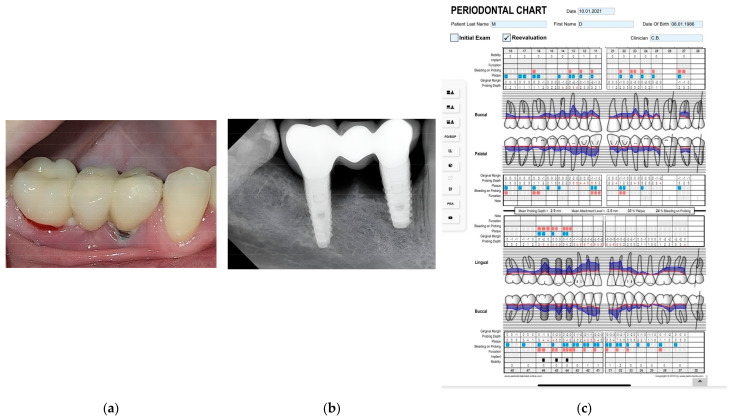
The peri-implantitis of a 36-year-old female at 5 years from implant placement: (**a**) intraoral aspect, (**b**) radiological aspect, (**c**) periodontal chart of the patient.

In all cases, clinical and radiological examinations were performed (Figure 1a,b)

During the clinical examination, the following parameters were determined: periodontal pocket depth of the natural teeth present in the oral cavity to evaluate the periodontal status of the patients, peri-implant pocket depth, Löe–Silness gingival index, peri-implant mucosal thickness, and peri-implant keratinized mucosal width. The same periodontology specialist evaluated all the above parameters. Intraoral photographs of the implant site were taken, and a ten-question questionnaire was completed. The questionnaire was used to collect data on the patient’s experience with the implant, and the instruments and methods used to clean the areas around the implants.

The periodontal status of the existing natural teeth was assessed by determining the depth of the periodontal pocket at 6 points around each tooth: mesio-buccal, buccal, disto-buccal, mesio-oral, oral, and disto-oral points, using the William probe (Hu-Friedy Italy Srl, Milano, Italy), which contains gradations from 1 to 10 mm, omitting the 4 and 6 mm gradations for better operator orientation (Figure 1c).

Similarly, the peri-implant pocket depth was evaluated in the same 6 points using a plastic periodontal probe (Hu-Friedy Italy Srl, Milano, Italy) with gradations from 1 to 15 mm (Figure 1a).

Using the Löe–Silness gingival index, we evaluated the condition of the gingiva, i.e., the presence and degree of peri-implant mucositis, as follows:

0—No visible inflammation.

1—Slight discoloration, without gingival bleeding.

2—Obvious inflammation, gingival bleeding appears a few seconds after probing.

3—Visible gingivitis and spontaneous gingival bleeding [29].

Regarding the thickness of the peri-implant mucosa, it is essential to know that two final phenotypes can be distinguished. One extreme is thin, wavy mucosa; the other is thick mucosa with a flat bone margin. To evaluate the peri-implant mucosal thickness, a plastic periodontal probe was inserted under the gingival margin: a value of 0 was taken for thin mucosa if the probe penetrated the gingiva; a value of 1 was registered for thick mucosa if the probe did not penetrate the gingiva [29].

The width of the peri-implant mucosa was determined in mm by placing the periodontal probe centrally between the muco-gingival junction and the most coronal point where the attached mucosa emerges [29].

**Figure 2:**
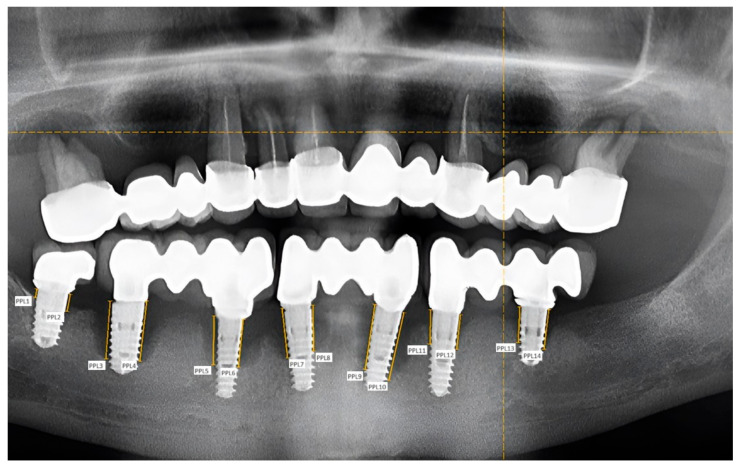
Lower arch: measurement points on the mesial and distal surfaces of the implants—61-year-old male patient; implant placement: 11 years ago.

The panoramic radiographs were then evaluated and measured. Measurements were performed using the two-dimensional image analysis method with the Image-Pro Insight 9.3 program (Media Cybernetics, Rockville, MD, USA). After calibrating the images, measuring points were taken on the mesial and distal sides of the implants, at the junction of the bone surface and the mucosa. Then, the amount of bone resorption was measured in millimeters (Figure 2).

Data were collected using Excel 2019, statistical analysis was performed using GraphPad Prism 9.5.1 (GraphPad Software, Boston, MA, USA), outliers were filtered using the Grubbs test, data distribution was examined using the Kolmogorov–Smirnov test, and groups were compared using ANOVA and t-tests. The significance level was set at 0.05.

## 3. Results

Overall, 15% of the cases examined were in the under-35 age group, 52.5% were in the 35–55 age group, and 32.5% were in the over-55 age group. Females and males were almost equally represented in all age groups.

The degree of bone resorption was first examined by age group. In the under-35 age group, the amount of bone resorption was 3.08 ± 0.03 mm; in the 35–55 age group, it was 3.09 ± 0.04 mm; and in those over 55 years, it was 2.05 ± 0.09 mm. Statistically, there was no significant difference in the degree of bone resorption between the age groups (*p* = 0.62). A similar result was obtained according to the time elapsed since implant placement. In implants placed 1–3 years ago, the degree of bone resorption was 2.82 ± 0.04 mm; in implants placed 4–7 years ago, it was 3.39 ± 0.12 mm, while in implants older than 7 years it was 2.08 ± 0.05 mm. Statistically, there were no significant differences between the groups (*p* = 0.57). More bone resorption can be observed in the first years, which does not necessarily worsen in the future.

No statistically significant differences were found between genders (*p* = 0.71), although a slightly higher degree of bone resorption was observed in females (3.4 mm ± 0.15) than in males (2.45 ± 0.07 mm ).

**Figure 3:**
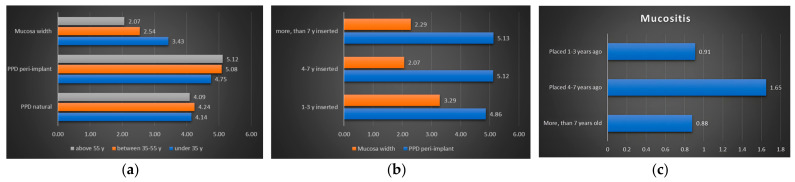
The changes following peri-implant parameters: (**a**) periodontal pocket depth changes reported for age groups; (**b**) the width of the keratinized mucosa compared with the peri-implant periodontal pocket depth according to the time elapsed since the implant placement; (**c**) average values of Löe–Silness gingival index as a function of the time elapsed since implant placement.

The periodontal status of the patients was evaluated by measuring the periodontal pocket depths around the natural teeth present in the oral cavity (Table 1 and Table 2) (Figure 3a,b). The periodontal pocket depth around implants showed significantly greater values (*p* = 0.0006) than natural teeth.

From a statistical point of view, the peri-implant periodontal pocket depth did not show a significant difference between age groups (*p* = 0.78), or in the time elapsed since implant placement (*p* = 0.75).

The comparison of the PPD (periodontal pocket depth) values of the natural teeth with the peri-implant probing depth values is shown in Table 1 and Table 2. These values were compared within the different age groups. In the group under 35 years of age, there is no significant difference between the probing depth values around the implant and the PPD around the natural teeth of the same person.

For the 35–55 and over-55 years age groups, the peri-implant probing depths were significantly higher than the PPD values of the natural teeth of the same person in each group concerning the time elapsed since implant placement. The mean values of PPD and peri-implant probing depth and the corresponding *p* values are shown in Table 1 and Table 2.

The highest values for the Löe–Silness gingival index were found for the 35–55 age group and implants placed 4–7 years ago (Figure 3c), indicating status between level 1 and 2 inflammations. The values for the 35–55 age group show a significant difference compared to those of the other age groups (*p* = 0.04). However, no statistically significant differences were found between the other groups (*p* > 0.05). We found no differences between the genders in the degree of inflammation (*p* = 0.45).

Both mucosal thickness and keratinized mucosal width (*p* < 0.0001) showed significant differences compared with peri-implant periodontal pocket depth (Figure 1a and Figure 3a). There was a tendency for an inverse relationship between the thickness and width of the keratinized mucosa and the peri-implant periodontal pocket depth, with the mucosal parameters increasing as the peri-implant periodontal pocket depth decreased.

There were no significant differences in mucosal thickness between age groups (*p =* 0.06) or time since implant placement (*p* = 0.106) (Figure 1a).

The width of the keratinized mucosa was not affected by age (*p* = 0.24) or time since implant placement (*p* = 0.15). The mean values of mucosal thickness around implants according to age groups and time since implant placement are highlighted in Table 1 and Table 2.

Based on the questionnaires, the most frequent subjective symptoms experienced by people with implants were collected: the most frequent was the squeezing of food next to the implant (22.5% of the respondents reported it), followed by bleeding gums (20%), the visible perception of receding gums (in 17.5%), and, to a lesser extent, halitosis and the visibility of the metal border along the gingival margin as perceived discomforts. All patients reported receiving information on how to clean the area around the implant after the treatment. In addition, they learned about this topic from friends and the Internet. In addition to the toothbrush, most of the patients used mouthwash (77.5%), dental floss (35%), and interdental brushes (32.5%) as additional methods; some of them used water- flossers (25%), and the fewest used a single-bristle toothbrush (10%) and super floss (10%) as additional oral hygiene products.

## 4. Discussion

Dental implantation is a modern method of tooth replacement. Its technology is constantly developing, but this treatment method can still have many complications. The dentist, the patient, or the implant system itself may cause unintended consequences. Complications can be intraoperative (e.g., nerve injury, sinus opening—especially with sinus lift) or postoperative (e.g., implant exposure, inflammation, bone resorption) [30].

The present study evaluated the peri-implant complications related to age, gender, time elapsed since implant placement, periodontal health, mucosal thickness and width, subjective symptoms, and the oral hygiene habits of the patients. According to our findings, the age and gender of the patients do not influence bone loss around implants, but it can occur slightly more in females and 4–7 years after implant placement. Other studies from the literature support our findings related to the gender of the patients [31] but highlight a high correlation between bone loss and the patient’s age [32].

According to the “Consensus Report of the Sixth European Workshop in Periodontology,” the incidence of peri-implantitis is 28–56%, while that of peri-implant mucositis is around 80%. Risk factors include periodontitis, smoking, poor oral hygiene, and diabetes [13,33,34]. The patients included in this study were non-smokers, had good or acceptable oral hygiene, and had no systemic diseases.

The biological causes of complications include bacterial invasion of the peri-implant tissues, leading to inflammatory changes in the soft tissues and rapid bone destruction. In addition, personal factors, tissue deficiencies, systemic factors (osteoporosis, smoking, mental illness, diabetes mellitus, alcohol consumption), and biomechanical and overload factors may also lead to the development of complications [33,35]. Our study only included patients who did not have any pre-existing systemic disorders, which could have potentially affected the outcomes of our research. We were interested in studying the implant’s behavior and survival rate in intraoral conditions without being influenced by systemic diseases. This topic could be the subject of another study.

According to Ferreira’s study, the incidence of peri-implantitis and peri-implant mucositis is influenced by smoking and the thickness of the keratinized mucosa. A keratinized mucosa thickness of less than 2 mm significantly increases the incidence of inflammation around the implants [36]. In this research, the smoking factor was eliminated due to the protocol of the dental clinic: the surgeon refuses to place implants in smoking patients. The average width of keratinized mucosa around dental implants placed 1–3 years ago was more than 3 mm, associated with a peri-implant pocket depth of less than 5 mm, and around implants older than 4 years the average width of keratinized mucosa was less than 2.3 mm, associated with a peri-implant pocket depth of more than 5 mm. Our study was consistent with Ferreira’s findings: the lower the keratinized mucosal thickness around implants, the higher the incidence of peri-implant inflammation. Peri-implant mucositis precedes the development of peri-implantitis, so mucositis occurs in a much higher percentage than peri-implantitis itself. This is also supported by data from the literature [32,37,38]. An important clinical feature of prosthodontic consideration is the restoration’s fixation type: there can be screw-retained or cemented prosthetic works. All prosthetic works in the present research were cemented following the dental clinic’s protocol, resulting in a more esthetic appearance. Both methods have their advantages and disadvantages. Cemented restorations without an early and periodical screening can lead to early bone loss around implants with subgingivally placed restorations because of the undetectable excess cement [39]. Properly selecting the cement and the adequate, individualized treatment protocol can significantly decrease the risk of peri-implant bone loss [40]. Screw-retained bridges have a better long-term prognosis compared to cemented ones because of the missing excess cement on the cervical third; they are more feasible, and they can be removed for any future adjustment or repair, which would not be possible in the case of classic cemented restorations. However, the latter is more esthetic as the access hole is absent on the occlusal area. It allows for some angulation of the underlying implants [41,42,43,44].

Hard and soft tissue changes around the implant are multifactorial. They may be caused by trauma, periodontitis, anatomical conditions, thin mucosa, lack of keratinized mucosa, incorrect implant placement, tooth migration, and systemic diseases [45]. Another study demonstrated that the risk of developing peri-implantitis increases with smoking and the presence of the TNFα-308 GA/AA genotype [46]. Patients with background periodontal disease have a higher risk of developing peri-implantitis or peri-implant mucositis [47]. During this study, significantly greater PPD was observed around implants compared to natural teeth, which was related to the age of the examined patients and the time elapsed since implant placement. Only the under-35 age group and those with implants placed 1–3 years ago showed similar values. Periodically monitoring periodontal health can minimize the risk of peri-implant complications. This study’s results agree with other findings in the literature [48,49]. In patients with periodontal disease, bone loss and periodontal pocket depth around implants were greater than around natural teeth, without significant differences related to age or gender.

The leading cause of inflammation around the implants is bacterial colonization at the head and body of the implant. Therefore, perfect epithelial attachment is one of the fundamental factors for the long-term survival of the implants. Developing this epithelial attachment at the mucosal level also depends on the establishment of perfect cleaning of the implant head [25]. Each patient in this study who underwent implant placement in our dental clinic signed an informed consent form and committed to using the complementary oral hygiene methods previously presented by the surgeon. Another study is planned to evaluate the effects of using different adjunctive oral hygiene methods around dental implants.

Long-term experience in the dental implant literature indicates that a positive papillary bleeding index, a probing depth greater than 4 mm, and a slight bone loss in most cases reflect dental implants that are still functioning well, considering that healing after dental implant placement is the result of a foreign body reaction, with the formation of scar tissue. Therefore, measuring the probing depth and determining the papilla bleeding index in evaluating periodontal status around implants may lead to overdiagnosis and possibly overtreatment of suspected biofilm-mediated peri-implantitis conditions. The authors of the study state that treatment should be initiated only when clinical symptoms (discomfort, pain, swelling, discoloration, and the presence of pus) and significant bone loss over time, as confirmed by radiographs, are present. The goal of treatment should be to eradicate the infection, which may include implant removal [50].

As the loss rate of placed implants has increased, specific follow-up protocols have been established for daily practice. In the case of peri-implant mucositis, the diagnostics protocol includes a visual inspection that shows signs of inflammation around the implants (red color of the peri-implant mucosa, swollen soft tissues), significant bleeding (line or tear-shaped), the presence of pus on probing, deeper probing depths that exceed the standard, non-pathological range, and the absence of additional bone loss after initial bone remodeling [12,50]. In the case of peri-implantitis, the diagnostic protocol includes visible soft tissue inflammatory processes around the implants, a positive papilla bleeding index, pus on probing, deeper pocket depth compared to values measured at superstructure placement, and progressive bone loss after one year compared to previous radiographic bone level assessment. Without initial radiographs and previous probing depth values, radiographic evidence of 3 mm or higher bone loss and 6 mm or greater probing depth with significant bleeding suggests peri-implantitis. Assessment of the annual rate of bone loss might be helpful in daily clinical practice [11,14,50].

In epidemiologic studies, the same criteria should be used to determine the health of peri-implant tissues and detect peri-implant mucositis as in daily practice. The problem with epidemiologic studies is that radiographic and clinical information on suprastructure placement may not be available. Under these circumstances, the diagnosis of peri-implantitis requires 3 mm or more from the implant platform to the bone contact and a positive bleeding index [12,50,51].

Although the findings of the current study are consistent with those mentioned earlier, it is crucial to acknowledge the limitations. The small sample size is an important constraint. Further studies with larger cohorts and standardized evaluation protocols are needed. This will provide a more comprehensive understanding of peri-implant disease. Moreover, additional research is needed to evaluate the influence of various factors such as different types of tobacco, implant-supported prostheses, adjunctive oral hygiene methods, and systemic diseases on dental implants’ longevity and survival rate.

## 5. Conclusions

In the cases examined in this study, there were no statistically significant differences between the time since implant placement and the degree of bone resorption: a large amount of bone resorption can be detected from the first years, similar to implants placed ten years ago. The degree of bone resorption, peri-implant periodontal pocket depth, and mucosal thickness and width did not differ significantly between genders and age groups. Within each group, the peri-implant probing depth was significantly greater than the periodontal pocket depth values of the natural teeth of the same individual concerning the time elapsed since implant placement. Periodontitis is a significant risk factor for the development of peri-implant inflammation, and patients with periodontitis had more significant bone loss and periodontal pocket depth around implants. In the population we studied, peri-implant mucositis was less common in the under-35 age group than in the 35–55 age group. Peri-implant inflammation can occur at any age, regardless of the lifetime of the implant.

## Figures and Tables

**Table 1:** Mean values of the measured parameters by age groups, with *p* values showing statistically significant differences. (PPD = periodontal pocket depth; PD = probing depth; SD = standard deviation.)

Age Group	PPD around Natural Teeth (Mean ± SD)	PD around Implants (Mean ± SD)	*p* Value	Peri-Implant Mucosa Width (Mean ± SD)
Under 35 years	4.14 ± 1.16	4.75 ± 1.32	0.188	3.43 ± 1.82
35–55 years	4.24 ± 0.83	5.08 ± 0.95	0.002	2.54 ± 1.32
Over 55 years	4.09 ± 0.91	5.12 ± 1.30	0.014	2.07 ± 2.10

**Table 2:** Mean values of the measured parameters grouped by the time elapsed since implant placement, with *p* values showing statistically significant differences. (PPD = periodontal pocket depth; PD = probing depth; SD = standard deviation.)

Insertion of the Implant (s)	PPD around Natural Teeth (Mean ± SD)	PD around Implants (Mean ± SD)	*p* Value	Peri-Implant Mucosa Width (Mean ± SD)
1–3 years ago	4.14 ± 0.88	4.86 ± 1.08	0.04	3.29 ± 1.52
4–7 years ago	4.09 ± 0.91	5.12 ± 1.30	0.014	2.07 ± 2.10
more than 7 years ago	4.27 ± 0.95	5.13 ± 1.03	0.016	2.29 ± 1.32

## Data Availability

The datasets analyzed during this study are available from the first author on request.

## References

[1] Block M.S. (2018). Dental implants: The last 100 years. J. Oral Maxillofac. Surgery.

[2] Buser D., Sennerby L., De Bruyn H. (2017). Modern implant dentistry based on osseointegration: 50 years of progress, current trends and open questions. Periodontol. 2000.

[3] Nervins M. (2014). Implant dentistry: A continuing evolution. Int. J. Periodontics Restor. Dent..

[4] Vajdovich I., Orosz M. (2012). Realization of tissue care concept by the use of Denti bone level implants. Three years of clinical experience in applying Denti BL implants. Fogorvosi Szle..

[5] Karthik K., Sivaraj S. (2013). Evaluation of implant success: A review of past and present concepts. J. Pharm. Bioallied Sci..

[6] Makary C., Menhall A. (2019). Primary stability optimization by using fixtures with different thread depth according to bone density: A clinical prospective study on early loaded implants. Materials.

[7] Cicciu M. (2020). Bioengineering methods of analysis and medical devices: Current trends and state of the art. Materials.

[8] Raghavendra S.J., Dhinakarsamy V. (2015). Osseoinegration. J. Pharm. Bioallied Sci..

[9] Iacono V.J., Bassir S.H., Wang H.H., Myneni S.R. (2023). Peri-implantitis: Effects of periodontitis and its risk factors—A narrative review. FOMM.

[10] Papaspyridakos P., Chen C.J., Singh M., Weber H.P., Gallucci G. (2012). Success criteria in implants dentistry: A systematic review. J. Dent. Res..

[11] Berglundh T., Armitage G., Araujo M.G., Avila-Ortiz G., Blanco J., Camargo P.M., Chen S., Cochran D., Derks J., Figuero E. (2018). Peri-implant diseases and conditions: Consensus report of workgroup 4 of the 2017 World Workshop on the Classification of periodontal and peri-implant diseases and conditions. J. Periodontol..

[12] Renvert S., Persson G.R., Pirih F.Q., Camargo P.M. (2018). Peri-implant health, peri-implant mucositis, and peri-implantitis: Case definitions and diagnostic considerations. J. Clin. Periodontol..

[13] Lindhe J., Meyle J. (2008). Group D of European Workshop on Periodontology. Peri-implant diseases: Consensus report of the Sixth European Workshop on Periodontology. J. Clin. Periodontol..

[14] Heitz-Mayfield L. (2024). Peri-implant mucositis and peri-implantitis: Key features and differences. Br. Dent. J..

[15] Heitz-Mayfield L., Salvi G.E. (2018). Peri-implant mucositis. J. Periodontol..

[16] Salvi G.E., Aglietta M., Eick S., Sculean A., Lang N.P., Ramseier C.A. (2012). Reversibility of experimental peri-implant mucositis compared with experimental gingivitis in humans. Clin. Oral Implant. Res..

[17] Hammerle C.H., Chen S.T., Wilson T.G. (2004). Consensus statements and recommended clinical procedures regarding the placement of implants in extraction sockets. Int. J. Oral Maxillofac. Implant..

[18] Derks J., Schaller D., Hakansson J., Wennström J.L., Tomasi C., Berglundh T. (2016). Peri-implantitis—Onset and pattern of progression. J. Clin. Periodontol..

[19] Kowalski J., Lapinska B., Nissan J., Lukomska-Szymanska M. (2021). Factors influencing marginal bone loss around dental implants: A narrative review. Coatings.

[20] Galindo-Moreno P., Catena A., Pérez-Sayáns M., Fernández-Barbero J.E., O’Valle F., Padial-Molina M. (2022). Early marginal bone loss around dental implants to define success in implant dentistry: A retrospective study. Clin. Implant. Dent. Relat. Res..

[21] Saravi B.E., Putz M., Patzelt S., Alkalak A., Uelkuemen S., Boeker M. (2020). Marginal bone loss around oral implants supporting fixed versus removable prostheses: A systematic review. Int. J. Implant. Dent..

[22] Robitaille N., Reed D.N., Walters J.D., Kumar P. (2016). Periodontal and peri-implant diseases: Identical or fraternal infections?. Mol. Oral Microbiol..

[23] Schwarz F., Derks J., Monje A., Wang H. (2018). Peri-implantitis. J. Clin. Periodontol..

[24] Genco R.J., Borgnakke W.S. (2013). Risk factors for periodontal disease. Periodontol. 2000.

[25] Zheng H., Xu L., Wang Z., Li L., Zhang J., Zhang Q., Chen T., Lin J., Chen F. (2015). Subgingival microbiome in patients with healthy and ailing dental implants. Sci. Rep..

[26] Ferreira S.D., Silva G.L., Cortelli J.R., Costa J.E., Costa F.O. (2006). Prevalence and risk variables for peri-implant disease in Brazilian subjects. J. Clin. Periodontol..

[27] Lee C.T., Huang H.Y., Sun T.C., Karimbux N. (2015). Impact of patient compliance on tooth loss during supportive periodontal therapy: A systematic review and meta-analysis. J. Dent. Res..

[28] Rokaya D., Srimaneepong V., Wisitrasameewon W., Humagain M., Thunyakitpisal P. (2020). Peri-implantitis update: Risk indicators, diagnosis, and treatment. Eur. J. Dent..

[29] Gera I. (2009). Periodontology.

[30] Causes and Treatment of the Failure of Dental Implant Restorations. https://www.semmelweis.hu/szajsebeszet.

[31] Dreyer H., Grischke J., Tiede C., Eberhard J., Schweitzer A., Toikkanen S.E., Glöckner S., Krause G., Stiesch M. (2018). Epidemiology and risk factors of peri-implantitis: A systematic review. J. Periodontal Res..

[32] Devi S., Duraisamy R. (2020). Crestal Bone Loss in Implants Postloading and Its Association with Age, Gender, and Implant Site: A Retrospective Study. J. Long-Term Eff. Med. Implant..

[33] Vajdovich I. (2017). Dental Implantology.

[34] Ogata Y., Nakayama Y., Tatsumi J., Kubota T., Sato S., Nishida T., Takeuchi Y., Onitsuka T., Sakagami R., Nozaki T. (2017). Prevalence and risk factors for peri-implant diseases in Japanese adult dental patients. J. Oral Sci..

[35] Dental Implants—Esthetic Complications. https://www.dental.hu/fogaszati-implantatumok-esztetikai-komplikaciok.

[36] Ferreira C.F., Buttendorf A.R., de Souza J.G., Dalago H., Guenther S.F., A Bianchini M. (2015). Prevalence of peri-implant diseases: Analyses of associated factors. Eur. J. Prosthodont. Restor. Dent..

[37] Mahardawi B., Jiaranuchart S., Damrongsirirat N., Arunjaroensuk S., Mattheos N., Somboonsavatdee A., Pimkhaokham A. (2023). The lack of keratinized mucosa as a risk factor for peri-implantitis: A systematic review and meta-analysis. Sci. Rep..

[38] Afrashtehfar K.I., Oh K.C., Jurado C.A., Lee H. (2023). Lack of keratinized mucosa increases peri-implantitis risk. Evid.-Based Dent..

[39] Staubli N., Walter C., Schmidt J.C., Weiger R., Zitzmann N.U. (2017). Excess cement and the risk of peri-implant disease—A systematic review. Clin. Oral Implant. Res..

[40] Kotsakis G., Zhang L., Gaillard P., Raedel M., Walter M., Konstantinidis I. (2015). Investigation of the Association Between Cement-Retention and Prevalent Peri-Implant Diseases: A Cross-Sectional Study. J. Periodontol..

[41] Rosenstiel S.F., Land M.F., Fujimoto J. (2016). Contemporary Fixed Prosthodontics.

[42] Chio A., Hatai Y. (2012). Restoration of two implants using custom abutments and transverse screw-retained zirconia crowns. Am. J. Esthet. Dent..

[43] Wittneben J.G., Joda T., Weber H.P., Bragger U. (2017). Screw retained vs. cement retained implant-supported fixed dental prosthesis. Periodontol. 2000.

[44] Jánosi K.M., Cerghizan D., Berneanu F.D., Kovács A., Szász A., Mureșan I., Hănțoiu L.G., Albu A.I. (2023). Full-mouth rehabilitation of a patient with gummy smile—Multidisciplinary approach: Case Report. Medicina.

[45] Hammerle C.H., Tarnow D. (2018). The etiology of hard- and soft-tissue deficiencies at dental implants: A narrative review. J. Periodontol..

[46] Petkovic-Curcin A., Zeljic K., Cikota-Aleksic B., Dakovic D., Tatic Z., Magic Z. (2017). Association of cytokine gene polymorphism with peri-implantitis risk. Int. J. Oral Maxillofac. Implant..

[47] Altay M.A., Tozoğlu S., Yıldırımyan N., Özarslan M. (2018). Is history of periodontitis a risk factor for peri-implant disease? A pilot study. Int. J. Oral Maxillofac. Implant..

[48] Lv P.-X., Zhong J.-S., Ouyang X.-Y., Iao S., Liu J., Xie Y. (2024). Investigation of peri-implant diseases prevalence and related risk indicators in patients with treated severe periodontitis over 4 years after restoration. J. Dent. Sci..

[49] Astolfi V., Ríos-Carrasco B., Gil-Mur F.J., Ríos-Santos J.V., Bullón B., Herrero-Climent M., Bullón P. (2022). Incidence of Peri-Implantitis and Relationship with Different Conditions: A Retrospective Study. Int. J. Environ. Res. Public Health.

[50] Coli P., Christiaens V., Sennerby L., de Bruyn H. (2017). Reliability of periodontal diagnostic tools for monitoring peri-implant health and disease. Periodontol. 2000.

[51] Araujo M.G., Lindhe J. (2018). Peri-implant health. J. Clin. Periodontol..

